# Management of Ankyloglossia and Breastfeeding Difficulties in the Newborn: Breastfeeding Sessions, Myofunctional Therapy, and Frenotomy

**DOI:** 10.1155/2016/3010594

**Published:** 2016-08-30

**Authors:** Elvira Ferrés-Amat, Tomasa Pastor-Vera, Paula Rodríguez-Alessi, Eduard Ferrés-Amat, Javier Mareque-Bueno, Eduard Ferrés-Padró

**Affiliations:** ^1^Service of Oral and Maxillofacial Surgery, Hospital de Nens de Barcelona, Barcelona, Spain; ^2^Service of Pediatric Dentistry, Hospital de Nens de Barcelona, Barcelona, Spain; ^3^Department of Oral and Maxillofacial Surgery, Faculty of Dentistry, Universitat Internacional de Catalunya, Barcelona, Spain; ^4^Service of Speech and Orofacial Myofunctional Therapy, Hospital de Nens de Barcelona, Barcelona, Spain; ^5^Service of Pediatrics and Service of Human Lactation, Hospital de Nens de Barcelona, Barcelona, Spain; ^6^Department of Oral and Maxillofacial Medicine and Oral Public Health, Faculty of Dentistry, Universitat Internacional de Catalunya, Barcelona, Spain

## Abstract

The problems of suction in newborns give rise to multiple consequences for both the mother and the newborn. The objective of this paper is to present a case of ankyloglossia (“tongue-tie”) and the suction problems that were treated by a multidisciplinary team. The subject is a 17-day-old male patient, with ankyloglossia and suction problems during breastfeeding (pain in the breastfeeding mother, poor weight gain, and long breastfeeds). The patient followed the circuit established in our centre between the services of Oral and Maxillofacial Surgery and Breastfeeding and Speech Therapy and Orofacial Rehabilitation (CELERE). The evolution following the breastfeeding sessions, the myofunctional stimulation, and the lingual frenotomy was very favourable, thereby solving the suction problems that the newborn presented. All our patients receive breastfeeding sessions and myofunctional therapy as treatment. We know that a frenotomy is not always necessary and we believe that the stimulation of sucking before and after the surgical intervention is important in order to improve the final result.

## 1. Introduction

Newborns can have breastfeeding difficulties for many reasons, for example, their immaturity (preterm infants, before 37 weeks), and on other occasions, it is caused by the premature separation of mother and child due to the hospitalisation of either or because the baby has lost its sucking reflex. In other situations, there is interference caused by teats or soothers or even poor nursing posture when breastfeeding which makes sucking difficult [1–3].

The limitation in lingual mobility in newborns is another one of the reasons that can compromise sucking and latching on to the breast during the breastfeed [2, 4–7].

Around 3% of infants are born with a short lingual frenulum which can lead to breastfeeding problems [6, 8, 9]. The most important problems are those related to poor weight gain, excessively long feeds, and nipple pain of the mother while breastfeeding [4, 6].

The lingual frenulum is a fibromucous membrane that joins the base of the tongue with the floor of the mouth, and when it is short and restricts movement, we term this ankyloglossia or tongue-tie. It consists of the remains of embryonic tissue, produced in the early stages of the development of the oral cavity. An incorrect division of the genioglossus and hypoglossal muscles is considered [4, 8, 10–12]. It seems to have a genetic aetiology and is mainly found in male infants [3, 13–16].

Hence, ankyloglossia is a limitation in lingual mobility thereby impeding tongue protrusion as well as the lifting of the tip of the tongue due to the shortness and/or the genioglossus muscles [4, 10–12].

The aim of this work is to present a case of ankyloglossia with sucking problems treated by the Suction Pathology Unit (CELERE). This multidisciplinary unit is incorporated in the Foundation of Hospital de Nens de Barcelona (FHNB) and is made up of the Service of Maternal Lactation, the Service of Speech Therapy and Orofacial Myofunctional Rehabilitation, and the Service of Oral and Maxillofacial Surgery, which is responsible for the study, treatment, and follow-up of newborn patients that have sucking problems. It is a much debated topic, on which there is still quite limited scientific evidence.

## 2. Case Report

The case involves a 17-day-old newborn of masculine sex that attends the Suction Pathology Unit (CELERE), who was referred by his pediatrician as a result of sucking problems during breastfeeding and ankyloglossia. The diagnosis of ankyloglossia is carried out according to the Coryllos classification which defines four types of frenulum: Type I: fine and elastic frenulum, where the tongue is anchored from the tip to the alveolar ridge and it is found to be heart-shaped; Type II: fine and elastic frenulum, where the tongue is anchored from 2–4 millimetres of the tip to almost near the alveolar ridge; Type III: thick, fibrous, and not elastic frenulum; the tongue is anchored from the middle of the tongue to the floor of the mouth; Type IV: the frenulum cannot be seen, but when palpation is performed, one can feel fibrous anchoring and/or thick and shiny submucosa from the base of the tongue to the floor of the mouth [8, 17].

Besides the Coryllos classification, functional clinical criteria were also taken into account: poor weight gain (less than 100 grams per week), excessively long breastfeeds (more than 60 minutes), and maternal pain (maternal breastfeeding should not be a cause of pain for the mother).

The data has been gathered in three different moments of the treatment: at the beginning of it, at two weeks of our action, and at the end of the treatment, and the degree of ankyloglossia, maternal pain during breastfeeding, weight progress, and the length of the feeds were evaluated 15 days after.

The Visual Analogue Scale (VAS) was used in the evaluation of the pain [18].

The evolution of weight gain is assessed through weighing the child before each breastfeeding and stimulation session, always keeping in mind using the same scales (digital).

The time of the breastfeeds was measured by the mother herself, and to this end, the following time intervals were established: <15 minutes, 15–30 minutes, 30–60 minutes, and >60 minutes; the value chosen for the study and evaluating the evolution was the highest time interval.

The case involves a 42-year-old mother, without previous pregnancies. It was a natural birth from 39 weeks' gestation and a cesarean delivery was performed due to the hypertension of the mother. The weight of the child at birth was 2.900 grams, and the 1-minute APGAR score was 7 and the second test, performed 5 minutes later, was 9, without peri- or postnatal complications. The type of lactation chosen was exclusive breastfeeding.

**Figure 1:**
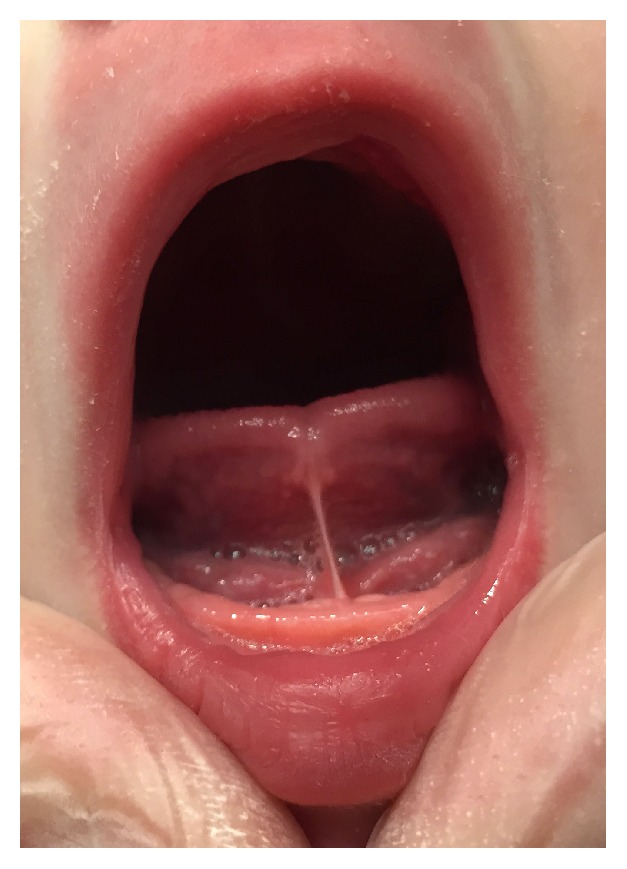
Lingual frenum with degree II ankyloglossia.

The weight of the newborn on the first visit was 2.970 grams. Maternal pain during breastfeeding was 10 (VAS). Poor weight gain was recorded: less than 100 grams per week. The breastfeeds were excessively long: more than 60 minutes. A thorough physical examination of the baby was undertaken with special attention to muscle tone and the anatomy of the oral cavity and the maxillofacial region. The newborn was diagnosed with ankyloglossia degree II (Coryllos classification) by visual inspection and palpation: slight physiological retrognathia (normal condition of mandible development, considering the age), poor latching, and unproductive sucking (Figure 1).

The treatment began through undertaking breastfeeding sessions, in order to correct nursing posture and improve latching on to breast. At the same time, work with a specialist in orofacial rehabilitation was done involving the stimulation of sucking and rooting reflexes and the carrying out of intraoral and extraoral exercises.

The extraoral stimulation exercises are aimed at improving the newborn's rooting reflex which are exercises that stimulate the masseter muscle (putting pressure with the index fingers and thumb, in a circular form in the area of the masseter muscles), stimulating the rooting reflex in the perioral region (with the thumbs and index fingers, moving forward on the upper and inferior lip in an alternating way and also making movements around the lips and on them with the fingers). The intraoral exercises have the function of stimulating the sucking reflex of the newborn. The areas to be stimulated are the palate, tongue, the inner surface of the cheeks, and the sucking reflex itself (through rotational movements while the newborn sucks the index finger) [19, 20].

It was recommended to the family to carry out the exercises a minimum of 3 times a day, repeating it 6 times on each occasion. This is always best recommended before the feeds as the baby has more appetite and is more likely to be cooperative. It is also suggested to maximise hygiene measures before beginning stimulation and/or use sterile gloves. The myofunctional therapy sessions will have a duration of 20 minutes and they are carried out twice a week during a one-month period, followed by the breastfeeding sessions [20, 21].

In the joint examination of the case, it was decided to indicate the performing of a lingual frenotomy which the mother accepted. It was undertaken on the 21-day-old infant in the surgery area of the hospital.

**Figure 2:**
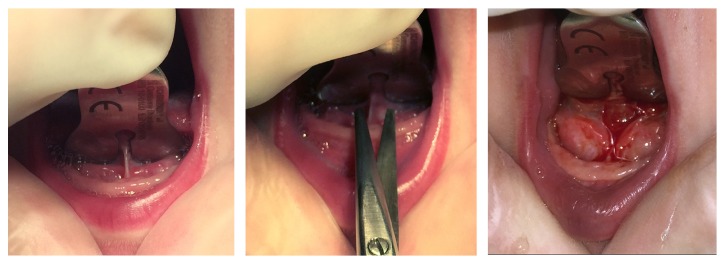
Lingual frenotomy, surgical technique.

A grooved probe and 12-centimetre-long Metzenbaum dissecting scissors were used (Figure 2). Following surgery, functional stimulation was carried out and immediately followed by breastfeeding.

The first check-up was performed 17 days after the frenotomy (newborn was 5 weeks old). Maternal pain had reduced to 5 (VAS scale), and the weight of the baby increased 200 grams weekly, reaching a weight of 3.180 grams, and the time of the feeds reduced to 30 minutes.

A second check-up was carried out on the newborn at 9 weeks of age. The pain of the mother reduced to 0 (VAS scale), and the patient's weight continued to increase by 200 grams weekly. The weight was 4,600 kilograms and the time of the feeds reduced to 15 minutes. The patient was discharged.

**Figure 3:**
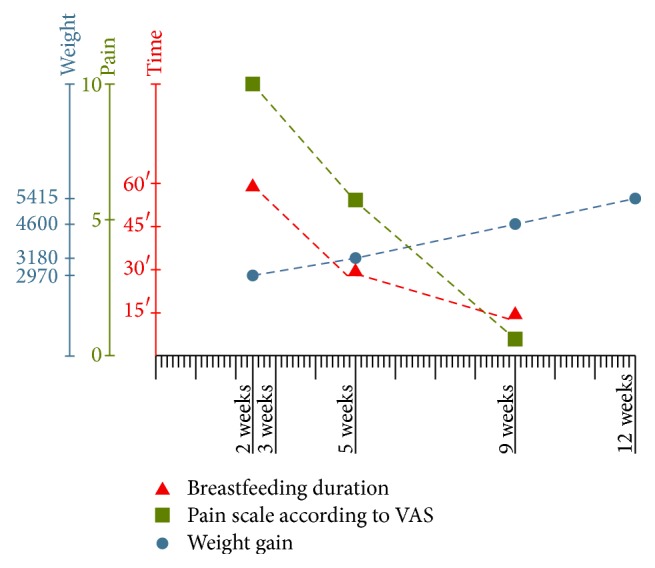
Graph of the progress of the patient following the CELERE circuit.

A graph has been prepared that summarises the monitoring of the patient's progress by following the circuit established by the Suction Pathology Unit at our hospital centre (Table 1 and Figure 3).

## 3. Discussion

The World Health Organization (WHO) has recommended that mothers exclusively breastfeed for the first 6 months of the child's life [22]. The benefits of breastfeeding and the reasons of early weaning have been widely studied. A short lingual frenulum has been considered as one of the problems of breastfeeding, although it is still a much debated theme among health professionals [1, 3, 23–26].

The exploration of the oral cavity must be systematic in the examinations of newborns and infants, focusing on the presence of ankyloglossia, without overlooking its detection [3, 8].

Coryllos only used anatomical criteria, while Hazelbacker includes valid functional criteria. Validation studies have been carried out in recent years with reproducible items that are both anatomical and functional [6, 7].

A lingual frenulum is a normal anatomical finding whose insertion point and Coryllos classification are not correlated with breastfeeding difficulties [27]. We use the Coryllos classification in combination with breastfeeding challenges.

Currently, there is a debate on how best to manage young infants with tongue-tie who have breastfeeding problems [5, 7, 26, 28].

Amir et al. in 2006 affirmed that the classification of Hazelbacker is highly reliable in the recommendation of a frenotomy in newborns [6]. Martinelli et al. in 2015 demonstrated that, after a lingual frenotomy, babies with a short lingual frenulum show changes that favour the appropriate standards of breastfeeding and all the symptoms reported by the mothers improved [2]. Furthermore, Power and Murphy in 2015 conclude that 50% of the breastfed babies with ankyloglossia will not encounter any problems [5].

In our protocol, we stimulate sucking and rooting reflexes before frenotomy, in cases of tongue-tie, to improve the orofacial function and we agree with Martinelli et al. who in their histological study said that the frenulum with ankyloglossia had significant number of striated skeletal muscle fibers. The high amount of type I collagen fibers in deep areas may justify the restriction of the tongue movement. Due to this fact, lingual frenectomy may be considered the appropriate procedure to release the tongue in order to provide better oral functions [12].

The evolution of our patient, after the breastfeeding sessions, orofacial stimulation, and the frenotomy, was very favourable, managing to address the sucking problems that the newborn baby presented. It improved through eliminating the pain of the mother; the times of the feeds were reduced, and the newborn managed to reach the ideal weight for his age.

The age of the patients is a decisive factor to achieve optimal results, and it is vital to act as soon as possible and in a multidisciplinary way. The collaboration and motivation of the family also play a decisive role in the resolution of unproductive suctioning in newborns, and it is essential to attend all the check-ups and programmed sessions and comply with the guidelines set out for newborns and accomplish all the tasks assigned.

## 4. Conclusions

Unproductive suction that some newborns have due to ankyloglossia and that occasionally causes pain for the mother during breastfeeding and the excessively long breastfeeds which determine the infant's weight gain can be resolved as a result of the action of a multidisciplinary team. A complete oral examination (morphological and functional) must be undertaken which includes the tongue, particularly when feeding problems exist. It is important to rule out abnormalities that can be the cause of sucking problems in breastfeeding.

It is recommended to firstly correct nursing posture during breastfeeding and stimulate sucking and rooting reflexes. Frenotomy is necessary upon the diagnosis of ankyloglossia, interfering in the breastfeeding, and when it is done, sucking stimulation is recommended before and after the surgical intervention.

In this clinical case, the multidisciplinary treatment has been a decisive factor that has resulted in the satisfactory evolution of our patient.

## Figures and Tables

**Table 1:** Table of the progress of the patient following the CELERE circuit.

	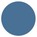 Weight	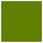 Pain	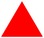 Time
2 weeks	2970	VAS 10	+60′
3 weeks	FRENOTOMY		
5 weeks	3180	VAS 5	30′
9 weeks	4600	VAS 0	15′
12 weeks	5414	VAS 0	15′
